# Participants and developers experiences with a chronic pain self-management intervention under development: A qualitative study

**DOI:** 10.1177/2050312118817427

**Published:** 2018-12-04

**Authors:** Kjersti Grønning, Torunn Hatlen Nøst, Toril Rannestad, Ola Bratås

**Affiliations:** Department of Public Health and Nursing, Norwegian University of Science and Technology, Trondheim, Norway

**Keywords:** Primary care, user involvement, stakeholders, health promotion, chronic pain, qualitative study, self-management

## Abstract

**Background:**

Non-pharmacological interventions aim to promote health and self-management for people with chronic pain.

**Objective:**

The aim of this study was to explore if the participants’ experiences with a self-management intervention under development were aligned with the developers’ rationale and desired outcome of the intervention.

**Methods:**

This was a qualitative study interviewing both participants and developers of a chronic pain self-management intervention. Seven participants, six females and one male in the age from early thirties to mid-seventies attended the chronic pain self-management intervention developed by the staff at a Healthy Life Centre. The data were analysed by the systematic text condensation method.

**Results:**

The analyses showed that the participants evaluated the intervention as valuable. They described using coping techniques to manage their chronic pain better, and the developers stated that the aim with the intervention was to provide the participants with coping techniques. The intervention was built upon the developers’ professional knowledge and experience in cognitive techniques, health theories, models for behavioural change, and service user involvement.

**Conclusion:**

This study found that the chronic pain self-management intervention was in concordance with theory of health promotion and empowerment. The participants experienced the intervention as targeting their resources, capacities, and fulfilling social needs, which aligned with the developers aim with the intervention. The participants found the intervention evocative; they learned new ways to manage their pain through theory/education, movement exercises, homework, and sharing their experiences with each other.

## Introduction

In recent years, both national and international authorities have acknowledged the necessity of incorporating health-promoting strategies into public health policies.^1^ Similar to other Western countries,^2^ Norwegian authorities have implemented a healthcare reform that transfers more services from specialized healthcare to primary healthcare. As part of this reform, new services in primary care are being developed, such as the Healthy Life centres (HLCs).^3^ HCLs are a part of the national strategy to promote healthy lifestyles, prevent lifestyle disease, and support people with health-related challenges to enhance their physical and mental health. People with chronic pain are one group who need support to manage their lives with a chronic condition.

The psychosocial and functional consequences of having chronic pain affect the individuals’ experience and impact of chronic pain^4,5^ in addition to socioeconomic consequences like early retirement, sick leave,^6,7^ and increased healthcare utilization.^7^ It is therefore important to acknowledge that psychological and social factors form an interactive complex of biopsychosocial processes that characterizes chronic pain. Alongside the psychological principles for treating chronic pain, there was a shift from viewing chronic pain purely within a ‘disease model’ to understanding chronic pain within a biopsychosocial perspective.^8^ As a result from the shifting perspectives, different non-pharmacological interventions became developed. In recent years, some non-pharmacological interventions are found effective^9–13^ while others have not.^14,15^ This disparity of results may be caused by the diversity and complexity of the interventions^12,16^ such as setting, content, and mode of delivery.^16,17^ However, a 2012 systematic literature review^18^ on effective delivery styles and content for self-management interventions for chronic musculoskeletal pain identified the following features as most essential: group setting, including a psychological component, healthcare professional as leaders or moderators of the programmes, and lasting a period up to 8 weeks. The authors argued that the group setting builds confidence and increases social interaction and integration into society, but more research was needed to explore the timing of exposure to chronic pain self-management interventions. Moreover, methodological research is needed to explore and isolate the interactions and effects of multicomponent therapies and complex interventions.^18^ The new Medical Research Council (MRC) guidelines for developing complex interventions^16^ state that the best practice is to develop interventions systematically. The MRC guidelines emphasize the importance of identifying the evidence base, selecting appropriate theory, and deciding the goal for the intervention when developing a new intervention.^19^

The guidelines for the HCLs state that their services should be based on knowledge of factors that promote health, emphasizing people’s resources and strengths^3^ which are key elements in health promotion.^20^ In health promotion, the salutogenic perspective focuses on salutary rather than risk factors, it looks at the entire person, not only the disease,^21^ and focuses on empowering people to increase control over and to improve their health.^20^ Empowerment-oriented interventions enhance wellness, provide opportunities for participants to develop knowledge and skills, and engage professionals as collaborators instead of authoritative experts.^22^ Empowerment is a process by which people gain control over their lives, where actions and activities make people feel strengthened.^22^

Although attempts have been carried out to explore functional details of self-management interventions for chronic pain,^12^ more research is needed to explore the effects, interactions, optimal means of delivery, and characteristics to inform future course design and improve outcomes.^18^ The MRC guidelines also suggest that interviewing stakeholders to optimize and examine uncertainties of an intervention prior to a full trial is recommended.^19^ The aim of this study was therefore to explore whether participants’ experiences with a chronic pain self-management intervention aligned with the developers’ rationale and desired outcome of the intervention under development.

## Methods

The setting for this qualitative study was an HLC in mid-Norway, which is a part of the public primary healthcare services. The HCLs offer interventions to people at risk of developing non-communicable diseases and people in need of support to carry out health behaviour changes or to cope with health-related problems or chronic conditions.^3^

As group interviews are suitable to facilitate interaction among people,^23^ three group interviews were conducted to explore the participants and developers’ experiences with the chronic pain self-management intervention under development. The inclusion criteria were as follows: (1) user of the HLC in mid-Norway, (2) self-reported chronic pain for 3 months or more, (3) having attended the intervention under development (entitled participants), or (4) being a health professional developing the intervention (entitled developers). There were no exclusion criteria. To recruit participants, the instructors of the intervention and the second author informed the participants about the purpose of the study; that participating in the interviews was voluntarily, and when the group interviews should take place (after the last session). The information was given orally and in writing. The developers were recruited to the group interview by an e-mail invitation.

The interviews were conducted from April to September 2015. The first and second author conducted the group interviews with the participants together, while the second author conducted the group interview with the developers alone. Both interviewers were females, had clinical backgrounds as nurses, and were experienced in conducting research interviews. The first author had both clinical and research experience in the field of patient education and self-management. The interviewers had no personal or professional relationships with the informants, but the first author had previously cooperated with one of the developers professionally. The interviews were held at the HLC and lasted from 40 to 70 min. Only informants and interviewers were present during the interviews. The interviews were audiotaped and transcribed verbatim.

Two thematic semi-structured interview guides were developed (Table 1). The interview guide to the participants contained questions about the participants’ reasons for attending the intervention, how they experienced the intervention, their reflections about the content, and how the intervention was delivered. The interview guide to the developers contained questions about why they developed the intervention, the role of the service user representative, and challenges in the developing process and in running the intervention. The first author wrote reflection notes after the interviews and used the notes during the analyses when discussing code groups and final categories.

**Table 1.** Interview guide.

Thematic interview guide – participants	Thematic interview guide – developers
• Rationale for participating Opening question: ‘Can you tell a little bit about why you wanted to participate in this intervention?’ Expectations • What they expected to learn and why The course • If they had participated in similar interventions before • Reflections about the intervention (number of participants, length, theory, movement exercises) • If they made use of the things they learned • What worked well/did not • Improvements	• Rationale for developing the intervention Opening question: *‘*Can you tell a little bit about why you decided to develop this course, the background?’ The developing process • How they cooperated, the role of the service user and the health professionals • Challenges • How and why did they choose the content (theory and exercises) Experiences with running the intervention • What worked well/did not • Group dynamics • Improvements

### The intervention

A description of the intervention is presented in Table 2, showing that the intervention contained a combination of education/theory, group discussions, and movement exercises. The education/theory focussed on chronic pain, the consequences of having chronic pain, how to manage the consequences of chronic pain, and how to cope with chronic pain (problem-solving, goal-setting, and coping techniques). The movement exercises aimed to improve balance, posture, and breathing and were based on Norwegian psychomotor physiotherapy.^24^

**Table 2.** Outline of the topics in the chronic pain self-management intervention.

Week/time	Theory	Exercises
1/13:00 – 15:30	Chronic pain • Aim of the course • Introduction to pain • How pain affects people’s everyday life	Movement exercises
2/13:00 – 15:30	Challenges • What stops me • My challenges and its consequences • My inner dialogue	Movement exercises
3/13:00 – 15:30	How to better cope with pain • What gives me energy • Self-confidence	Movement exercises
4/13:00 – 15:30	Personal goals • My goals • What do I want to achieve • Action plan	Movement exercises
5/13:00 – 15:30	I manage – I have choices • Coping techniques • Problem-solving	Movement exercises
6/13:00 – 15:30	The way forward • Repetition • How to move forward	Movement exercises

### Ethical considerations

Oral and written information about the purpose of the study was given to all informants. All informants signed a written consent before taking part in the interviews. The study was conducted in accordance with the Declaration of Helsinki,^20^ and the Norwegian Centre for Research Data approved the study (42056).

### Analyses

The data were analysed using systematic text condensation (STC),^25^ which is a modification of Giorgi’s phenomenological method. STC involves getting a sense of the whole material, discriminating meaning units, transforming and abstracting meaning units, and synthesizing meaning units into consistent statements. This method is suited for presenting experiences as the informants express them.^25^ NVivo, a data management programme, was used to systematize and code the data.^26^ The first author conducted the analyses in close collaboration with the last author. To get an overall impression of how the participants experienced the intervention as a whole, all transcripts from the participants who attended were read first. Then, the authors searched for text segments where the participants talked about their rationale for participating in the intervention, and their reflections about the content and delivery of the intervention (theory, group discussions, movement exercises, and potential improvements). During this step of the analyses, three preliminary themes were identified, followed by additional coding and establishing code groups. To ensure rigour of our analysis, the first and last author collaborated closely in the analysis process in addition to presenting preliminary findings at the forum ‘Health Promotion Research-an International Forum’.^27^ Discussions at the forum led to new ideas that were useful in the analyses process. In the final step of the analyses, the text was synthesized and reconceptualized into categories representing descriptions of how the participants experienced the intervention as a whole.

Then, the same procedure was conducted when analysing the interview with the developers. The final categories from the interviews with the participants were used as a starting point. The first author detected meaning units within the text, coded text segments, created code groups, and sorted the code groups into categories. The coding process included several re-readings, adjustments, and refinements through discussions between the first and the last author. Before finalizing the analyses, the findings were presented to the other co-authors who responded by e-mail.

## Results

Of 10 eligible participants, seven were interviewed. As participation in the study was voluntarily, no information about the participants who did not take part in the interviews was collected. The characteristics of the participants in the intervention are presented in Table 3, showing that the sample consisted of five females and two males, and their age ranged from early thirties to mid-seventies. Two participants stated that they had no pain or discomfort, one had slight pain or discomfort, two had moderate pain or discomfort, and one had severe pain or discomfort. The question regarding pain and discomfort is from the European Quality of Life Scale 5 Dimensions.^28^

**Table 3.** Participant characteristics.

Demograpic information	Numbers
Sex	
Female/male	5/2
Living situation	
Living alone/living with someone	5/1
Education	
Lower secondary school	1
Upper secondary school	4
Higher education (college or university)	1
Employment status	
Employed	1
Disability benfits	3
Partly employed/disability	1
Retired	1
Age group	
31–40 years	2
41–50 years	3
51–60 years	0
61 years or older	1
Pain/discomfort	
I have no pain or discomfort	2
I have slight pain or discomfort	1
I have moderate pain or discomfort	2
I have severe pain or discomfort	1
I have extreme pain or discomfort	0

Four of five informants participated in the interview with the developers; one was not available when the interview was scheduled. The sample consisted of physiotherapists and lifestyle coaches who worked full or part time at the HLC, and a service user representative.

**Figure 1.**
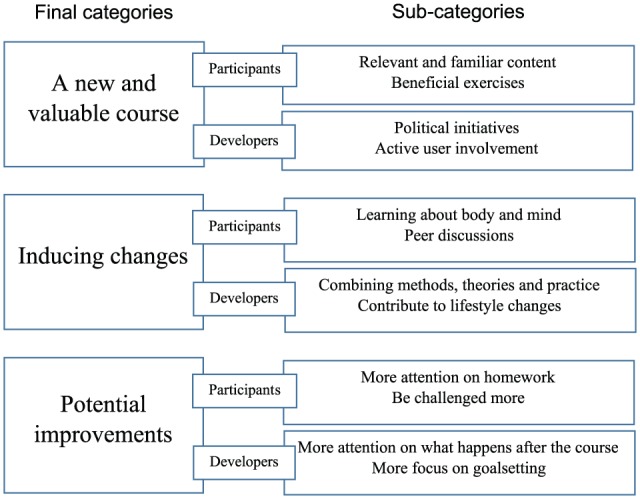
Categories.

The analyses showed that the participants found the intervention valuable. They mentioned several beneficial changes they had made to cope with their pain. One example was to use their bodies differently to alleviate pain. The participants were satisfied with the intervention as a whole, but had ideas that could improve the intervention. The developers’ aim with the intervention was to provide the participants with relevant coping techniques so they could cope with their pain and live good lives. Figure 1 presents an illustration of the final categories ‘A new and valuable course’, ‘Inducing changes’, and ‘Potential improvements’.

## A new and valuable course

The participants discussed their different experiences with the intervention. Some said they had never participated in an intervention like this before, while others had attended similar self-management courses at the hospital. They agreed that this intervention focussed on different ways to cope with chronic pain, but several said they had expected to learn more about coping, and that a great deal of the theoretical content was already known. They expressed, however, that the repetition was important, because they were reminded about things they had learned before, but had not always used. All participants expressed that this intervention was unique due to the combination of theory/education and movement exercises. The movement exercises were completely new to some, while one had tried similar exercises, and another had practised yoga. The participants all agreed that the movement exercises were very useful. Some explained that they had problems remembering the exercises and others needed some time before they understood the link between the theory/education and movement exercises. Below is an extraction of the discussion:

P1: It takes time to change … but we have got many nice exercises, if you only could remember them and make use of them in our everyday lives … that is the challenge.

P2: Yes, but we do not have a paper with the exercises on …

P1: No …

P3: We received a paper with the exercises.

P2: But did the paper contain the exercises?

P4: Yes, I have them.

(Participants, group interview 1)

The participants continued discussing the fact that the support from the group was helpful in many ways. Several said that they felt lonely due to their pain, but the support from the group helped, and they did not feel so lonely anymore. The participants also said they found it motivating to participate in the intervention, and they experienced the group as supportive and helpful in making changes.

When the participants discussed the content of the intervention, one participant, who had participated in similar interventions previously, said this group of participants had been more active compared to other interventions he had attended. The participants further agreed that the social fellowship and the size of the group were valuable because they made it possible to share experiences with each other.

P1: Something that has been very useful is the movement exercises and the ability to share experiences. That is always very useful. When we work with theory, it is easier that relevant experiences appear.

Interviewer: How many people have attended this intervention?

P1: I think we have been four persons in average.

P2: Yes, I think that is correct, four in average.

P1: We could preferably been more, but not too many.

P2: Yes …

Interviewer: How many would you prefer?

P1: Well, hard to say, perhaps 8-10? For me, the most valuable part was related to the theory, the ability to share relevant experiences and talk about them in relation to the theory, not just a presentation of the theory using a power point presentation.

(Participants, group interview 2)

When the developers were asked why they developed this self-management intervention, they said it was because people with chronic pain had few treatments options in primary care, and there was a need for interventions that could help people with chronic pain to live good lives despite their pain. They further explained that the content and form of the intervention was based on their previous professional experiences. A new feature they wanted to try was to combine theory/education with movement exercises. The developers said they considered the HLC as a suitable location for offering a chronic pain self-management intervention, since the aim of the HLC is to offer health promotion activities. The developers continued to explain that they decided to collaborate actively with a user representative because they had good experiences with this kind of collaboration previously.

D1: This [user involvement] is new to employees in the municipalities. It is actually new. We are not used to think about user involvement at a service level and in group- settings …

D2: It is very useful for us [health professionals] to get input from the user so we can adjust the content.

(Developers, group interview 3)

When the developers were asked about the aim of the intervention, they said their intention was to make a basic course, seeing this intervention as part of a process. After the intervention, they hoped the participants had learned enough to continue on their own, that they knew what to do and how to cope with their pain. The developers further said they selected the best from different theories on health behaviour as the theoretical platform. This choice was made in order to start an awareness process among the participants. The developers explained that they incorporated the movement exercises to make the participants feel and experience how their minds and bodies were linked together:

D1: In a way, we combine traditional HCL competency with learning and mastery methodology …

Interviewer: Do the rest of you agree? [Silence] …

D2: Hmmm

D3: Yes ….

(Developers, group interview 3)

## The course induced changes and gave a boost

The participants had different experiences with how the self-management intervention had led to changes in how they coped with their pain. The movement exercises were especially emphasized as useful because the exercises helped them to relax and calm down. By performing the movement exercises, several said they became more conscious about their body posture. One gave a concrete example about how she experienced less shoulder and neck pain because she had changed her body posture. The participants explained that they previously did not think about such exercises as a way of coping with pain. However, after practicing the exercises for a while, they saw it differently. They said, it was wonderful to learn how to use their body in a different manner.

P2: You can do some movement exercises that may help, but it is pro and cons if you are in great pain. You do not exactly look forward to start doing them, but they may help.

Interviewer: You talked about having less headache now?

P4: Yes, I figured out that I walked like a goose (laughter). I had not thought that (the body posture) had something to do with that before. It is the same case with my palms.

P1: I feel responsible. She was not present last time, and when I saw her, (demonstrating how she used to stand), we started to talk about how we used to posture. Normal people have their palms like this (pointing forward) and ours point in the opposite direction.

P2. Then we did some exercises, and suddenly, we became normal.

(Participants, group interview 1)

The participants also said the intervention made them more conscious about choosing activities that gave them energy instead of depleting their energy; they had learned to think differently about having chronic pain and to set realistic goals. Previous experiences of not reaching their goals made many of them feel sad. Through the discussions in the self-management intervention, the participants said they realized that their previous goals were unrealistic. Some were uncertain if they could manage on their own after the intervention was finished. Others stated that they felt motivated to continue working in order to manage their chronic pain better. One of the participants said:

I feel like I am about to start a new period in my life, and it is my responsibility what happens next. (Participant, group interview 2)

The developers discussed that they had consciously selected the content (both theory and movement exercises) to induce changes and provide the participants with coping techniques to manage their chronic pain better. To do so, the developers said they needed to focus on the participants’ mindsets and cognition and saw this focus as promoting healthy behaviours.

D1: We have discussed how an everyday life with chronic pain may be. Sometimes they wake up and feel all right and they want to do everything! However, the next day, they have to stay in bed because they are exhausted. When they carry out like this, they will not function with their family, in their everyday lives and in their social networks. I think that finding the right balance is about health promotion. To promote their health, to manage the balance. Finding the balance means to sometimes say «no», because you have to do that to have an okay day the day after. It is about sorting out and detecting one’s resources to carry out favorable changes in one’s life. In this intervention, it is a lot of health promotion by finding one’s resources; it is a lot of coping!

D3: Oh yes!

D2: Mmm, yes.

D1: It is difficult to say “no”. It is hard to find that balance. It is not health promotion when you, in a way, talk yourselves down and you do not manage your social life anymore.

(Developer, group interview 3)

When the developers were asked about the user representative’s role in the process of developing the self-management intervention, they agreed that a peer is crucial to ensure that the content is in line with the target group’s needs. They also said that a peer adds an extra dimension to the theoretical content. The health professionals were familiar with the theory of empowerment, coping, health behaviour changes, and self-management, while the layperson could verbalize how it feels to live and cope with chronic pain.

It’s advanced user involvement. A lay peer person needs to have a solution-oriented and resource-enhancing approach when talking about how to live and manage chronic pain. (Developer, group interview 3)

### Potential improvements

The participants agreed that they would recommend this self-management intervention to others. However, they had some suggestions that they thought would improve the intervention. The participants agreed that the intervention was too short and suggested that the intervention needed something more. One of the suggestions was to add an individual talk with the instructors before the group sessions:

It may be good to add an individual talk before the group sessions starts so the instructors could know a little bit more about us. (Participant, group interview 1)

Even though the instructors informed the participants that the self-management intervention was an introductory course, they said it was not enough to fully manage on their own. Some said they had been introduced to a lot of theory, but it was too much to grasp in only six weeks. In addition, they expressed that carrying out changes takes a lot of time.

When talking about other potential improvements, the participants said it was necessary for the instructors to pay more attention to the homework and give the participants more challenges. In the participants’ opinions, a thorough discussion of their homework assignments could make it easier to implement what they had learned in everyday life:

P1: I think the instructors should have challenged and activated us more […] we can manage; we have pain, but we are not fragile …

P2: I agree …

Interviewer: What kind homework have you had?

P3: Simple ones, how to set goals, concretize negative thoughts and such like.

(Participants, group interview 2)

When the developers were asked about potential improvements, they agreed that they needed to change several things before offering the self-management intervention to more people. They planned to focus more on goal setting during the entire self-management intervention, and they would concentrate more on discussing how the participants could manage on their own after the intervention. The developers felt that they had not prepared the participants well enough for the time after the self-management intervention, nor how the participants could manage on their own. They discussed that the participants could easily return to old habits if they were not prepared to manage on their own:

D1: It [the period after the intervention] must be in focus all the time. It may be wise to talk about this period a bit earlier, and not wait until the last session, since this is a process too …

D2: I agree …

D3: Me too.

(Developers, group interview 3)

Furthermore, the developers said they needed to better link the participants’ goal setting to what happens after the course. If they had done that, perhaps the participants would better know what areas they needed to work on. The developers also discussed if this self-management intervention could be adjusted to the participants’ individual needs. They agreed that individual adjustments would be nice, but found it challenging because the intervention was designed for a group of people and not individuals:

If they [the participants] for instance think they will be pushed on fitness or strength, that’s not exactly the goal in these groups. I think we need to take the average into account. (Developer, group interview 3)

## Discussion

The aim of this study was to explore whether participants’ experiences with a chronic pain self-management intervention were aligned with the goals and desired outcome of the intervention under development. The findings showed that the chosen theoretical foundation was relevant as the participants described changing mindsets and habits to better manage their pain and feel well.^21,29^ These descriptions support the importance of building complex self-management interventions upon relevant theory^16^ like health promotion.^3^ Empowering people to increase control over their health^21^ enhances wellness and provides participants with opportunities to develop necessary skills to handle their chronic pain,^22^ change habits, set realistic health goals, and choose the right strategies to realize those goals. The participants in this study made changes in how they handled their pain and became more actively engaged in taking care of their own health. Moreover, for some participants, taking part in the intervention became a substitute for a missing social network, which supports findings from another study.^11^

This study found that the theoretical basis for this self-management intervention is in concordance with health promotion^21,29^ and empowerment^22^ as the participants experienced the intervention as targeting their resources, capacities, and fulfilling social needs. The developers stated that they aimed to focus on the participants’ resources. The findings also indicate that self-management interventions for people with chronic pain may prevent loneliness and the risk of developing depression, which is important as depression and loneliness is shown to be challenging for this group of people.^30,31^ The participants further emphasized that sharing experiences about living with and managing chronic pain was very valuable. The benefit of sharing experiences is ponted to as essential in severeal studies of chronic pain^32,33^ and chronic conditions.^11^

As living with chronic pain influences social relationships, work life, and people’s everyday lives in general,^33^ developing self-management interventions that focus on resources, capacities,^31^ and empowering people to manage their everyday lives are essential. By exploring perspectives from both the participants and developers, the findings offer new insights into how self-management interventions may work^16^ as described by the participants in addition to the developers’ rationale for the intervention. Having several perspectives^16^ creates valuable knowledge about how health promotion and empowerment theory may work in clinical practice for people with chronic pain attending a self-management intervention. The MRC guidelines for complex interventions^16^ suggest that a systematic evidence synthesis is conducted when developing a new intervention. This intervention was based on the developers professionals’ knowledge, experiences, input from the service user, and designed as a combination of education/theory and movement exercises.^24^ Even though the findings did not reveal that a systematic evidence synthesis was conducted, the interviews clearly showed that the developers were conscious about the aim of the intervention, the theory, and how the intervention was supposed to work.

Both participants and developers suggested improvements to the intervention. The participants did not feel ready to manage on their own after the intervention, and the developers discovered that they needed to focus more on realistic goal setting. Helping participants with realistic goal setting and how to take care of their own health is essential in self-management interventions.^34^ Another suggestion from the participants that could improve the intervention was to add individual talks before the group sessions. The importance of individualizing and making participants feel involved in taking care of their health was pointed out as essential in a recent publication investigating user involvement in HLCs^35^ and how to help people make new choices and achieve changes.

### Strengths and limitations

The main strength of this study is that it explores perspectives from both participants and the developers of a self-management intervention. Including several perspectives from different stakeholders while developing a complex intervention is important.^16^ Different stakeholders’ perspectives generate new knowledge on whether the participants’ experiences with the intervention was in accordance with the developers’ goals for the intervention. However, some noteworthy limitations may hamper the findings, such as the small sample size and possible lack of information power. However, sufficient information power is also dependent on how narrow or broad the aim of the study is.^36^ As such, the aim of this study was narrow, and we were particularly conscious about asking probing questions to gather as much in-depth data as possible. In addition, the participants revealed both similar and varied experiences with the intervention. Another limitation is that we did not pilot test the interview guide, and therefore may have missed some important questions. We might also have recruited the most satisfied participants. The participants knew that this study explored a new intervention under development, and they may have been reluctant to share too negative experiences in case the municipality would not continue to offer this self-management at the HCL. In addition, we did not validate the findings by returning the transcripts to the participants for comments. The first author had previously cooperated with one of the developers, which could have influenced what the developers expressed during the interview. On the other side, both the participants and developers shared similar ideas to improve the intervention, indicating that they were honest and wanted to provide input to improve future interventions.

### Implications for clinical practice and research

The clinical implications from this study are that health professionals in charge of developing or delivering self-management interventions should consider combining theory/education with movement exercises in addition to focusing on what, and how to support the participants with coping techniques to feel better. Helping participants with realistic goal setting and time for sharing experiences are valued a great deal. Further research ought to explore more deeply how complex self-management interventions work to detect successful mechanisms that help people with chronic pain to manage their everyday lives. Randomized controlled trials accompanied by qualitative studies have a potential to investigate the effects of interventions in addtion to exploring the interactions between different components of the intervention. Such studies are warranted and will contribute with more evidence about what kind of interventions the healthcare services should develop to target the interactive complexity of biopsychosocial processes that characterizes chronic pain.

## Conclusion

This study found that the chronic pain self-management intervention was in concordance with theory of health promotion and empowerment. The participants experienced the intervention as targeting their resources, capacities, and fulfilling social needs, which were aligned with the developers aim with the intervention. The participants found the intervention evocative; they learned new ways to manage their pain through theory/education, movement exercises, homework, and sharing experiences with each other.
